# Supplementary figures and images for: Rapid Multiplex Small DNA Sequencing on the MinION Nanopore Sequencing Platform
Source: G3 (Bethesda). 2018 Mar 14;8(5):1649–57. doi: 10.1534/g3.118.200087 (PMC5940156; doi:10.1534/g3.118.200087)

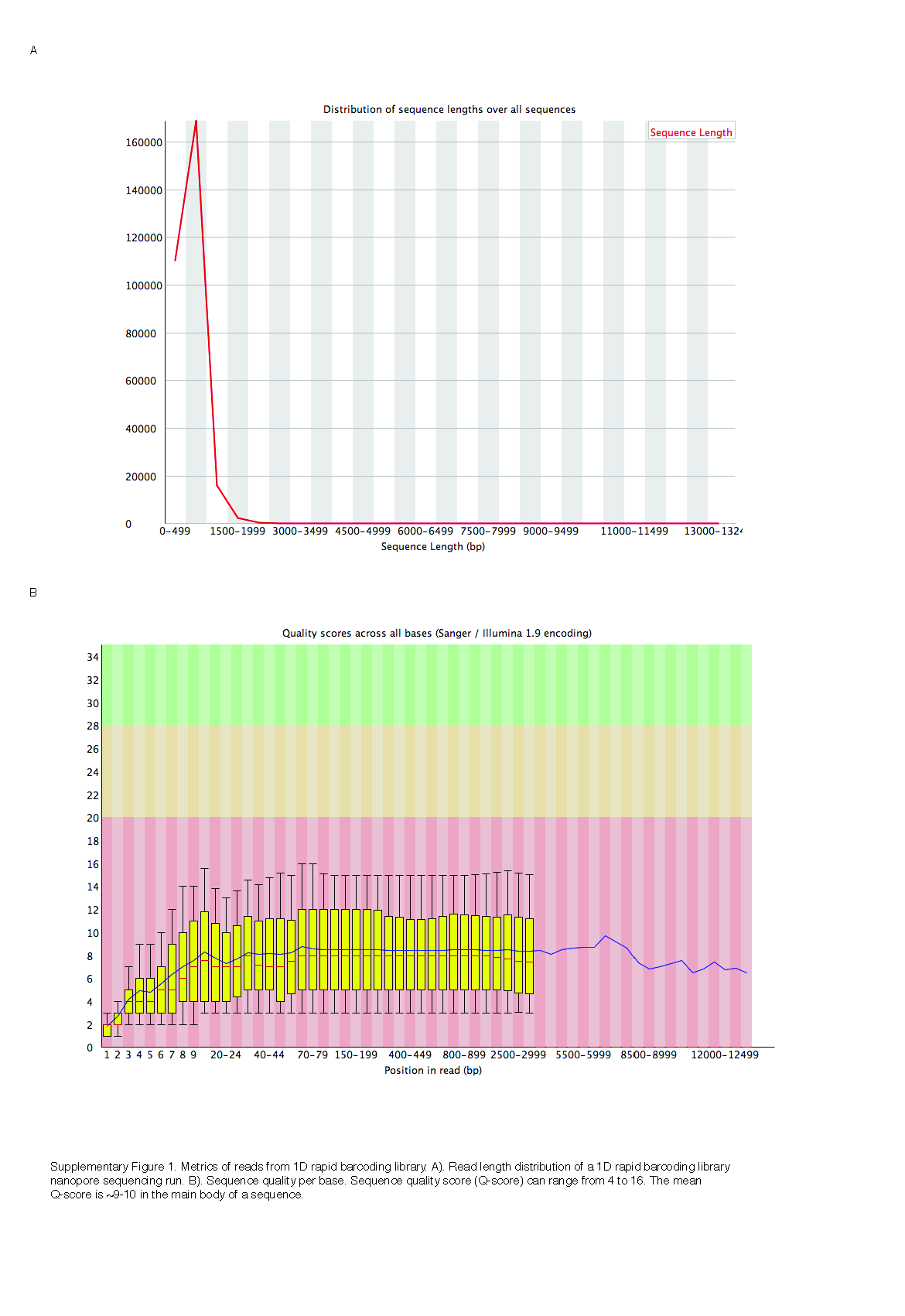

Supplement: Supplementary file 1 [file 1649FigureS1.tif]
