# Supplementary material for: Rapid Multiplex Small DNA Sequencing on the MinION Nanopore Sequencing Platform
Source: G3 (Bethesda). 2018 Mar 14;8(5):1649–57. doi: 10.1534/g3.118.200087 (PMC5940156; doi:10.1534/g3.118.200087)
Supplement: Supplementary file 2 [file 1649TableS1.docx]

**Supplementary Table 1. Sequence information**

MP1-6bp adaptor with 3’-ATTGCT overhang

Top: **5’-**GCTTGACATTCTGGATCGGTGACTGGAGTTCAGACGTGTGCTCTTCCGATCTATTGCT**-3’**

Bottom **3’AMINE**-TCTAGCCTTCTCGTGTGCAGA-**P-5’**

ME-6bp adaptor with a 3’-AGCAAT and 5’ blunt-end

Top: **5’-P-**CGTTGCAGCAGATGTGTATAAGAGACAG**-3’**

Bottom: **3’-**TAACGAGCAACGTCGTCTACACATATTCTCTGTC**-P-5’**

MP1-T adaptor with 3’-T overhang

Top: **5’-**GGAAGCTTGACATTCTGGATCGGTGACTGGAGTTCAGACGTGTGCTCTTCCGATCTT**-3’**

Bottom: **3’AMINE-**TCTAGCCTTCTCGTGTGCAGA**-P-5’**

ME-A adaptor with a 3’-A and 5’ blunt-endL

Top: **5’-P-**AGATGTGTATAAGAGACAG**-3’**

Bottom: **3’-**ATCTACACATATTCTCTGTC**-P-5’**

Control fragment sequence, 204bp

**5’-**CGGAGGCACTTCAATTTAACAGAAAAAACAATCATGTAAATAGAAAACCAGAGTGTCCTATCTGATAAGACTGATGGAAGCCTATTGAAAATAATAGAGCTTCTCCACTAATTTGGTTGACCGATTCATTTCCTGAATGAGTAATCCTCATCTTTGCCTGCCTTTACTTTAGCCAAGAGAAAGGTCCTTCTTGCCTGGCTCCCT

Control fragment sequence, 434bp

**5’-**CAGGAAACAGCTATGACCATGATTACGCCAAGCTATTTAGGTGACGCGTTAGAATACTCAAGCTATGCATCAAGCTTGGTACCGAGCTCGGATCCACTAGTAACGGCCGCCAGTGTGCTGGAATTCAGGCAAGCAGAAGACGGCATACGAGATCGTGATGTGACTGGAGTTCAGACGTGTGCTCTTCCGATCTCTGCACAATGTGCACATGTACCCTAAAACTTAGAGTATAATAAAAATAAAAAATAAAAAAAGAAGTCCAAAAAAAGATCGGAAGAGCGTCGTGTAGGGAAAGAGTGTAGATCTCGGTGGTCGCCGTATCATTCCTGAATTCTGCAGATATCCATCACACTGGCGGCCGCTCGAGCATGCATCTAGAGGGCCCAATTCGCCCTATAGTGAGTCGTATTACAATTCACTGGCCGTCGTTTTAC

M13F (-20) primer

**5’-** GTAAAACGACGGCCAG

M13R primer

**5’-** CAGGAAACAGCTATGAC
