# Supplementary material for: Rapid Multiplex Small DNA Sequencing on the MinION Nanopore Sequencing Platform
Source: G3 (Bethesda). 2018 Mar 14;8(5):1649–57. doi: 10.1534/g3.118.200087 (PMC5940156; doi:10.1534/g3.118.200087)
Supplement: Supplementary file 3 [file 1649TableS2.docx]

Supplementary Table 2. Comparison of software performance.

| **Software** | **Parameter** | **Node** | **Total cores used** | **Time** | **UA*** | **UA%** |
| --- | --- | --- | --- | --- | --- | --- |
| Pblat | -minIdentity=80 -minScore=40 -tileSize=10 -maxIntron=500 | 4 | 96 | 19m17.451s | 18,385 | 91.93 |
| minimap2 | -ax map-ont -k10 -O1 -E2 | 1 | 2 | 6m30.174s | 17,001 | 85.01 |
| minimap2 | -ax map-ont -k9 -O1 -E2 | 2 | 24 | 7m23.825s | 17,284 | 86.42 |

20,000 sequences from sample1 in run 2 were used to estimate the performance of software.

*UA, unique alignment. In pBlat, UA is defined as reads with only one alignment passing the pslReps filter -minCover=0.40 -minAli=0.80 -nearTop=0.001 –singleHit. In minimap2, UA is defined as reads with alignment score q=60 in the resulting sam file.
