# Supplementary material for: Rapid Multiplex Small DNA Sequencing on the MinION Nanopore Sequencing Platform
Source: G3 (Bethesda). 2018 Mar 14;8(5):1649–57. doi: 10.1534/g3.118.200087 (PMC5940156; doi:10.1534/g3.118.200087)
Supplement: Supplementary file 4 [file 1649TableS3.docx]

Supplementary Table 3. MinION karyotype assay results

| Run1-B01 |  |  |  |  |  |
| --- | --- | --- | --- | --- | --- |
| **Chr** | **UA** | **%UA** | **Adjusted Relative Copy Number** | **Modified Z-score** | **Abnormal?** |
| chrMT | 11 | 0.0012 | 0.7977 | -2.2545 | FALSE |
| chrX | 427 | 0.0474 | 1.9353 | 10.4218 | TRUE |
| chrY | 21 | 0.0023 | 0.3495 | -7.2486 | TRUE |
| chr1 | 710 | 0.0789 | 0.9636 | -0.4051 | FALSE |
| chr2 | 786 | 0.0873 | 1.0350 | 0.3897 | FALSE |
| chr3 | 591 | 0.0657 | 0.9524 | -0.5307 | FALSE |
| chr4 | 577 | 0.0641 | 0.9650 | -0.3895 | FALSE |
| chr5 | 533 | 0.0592 | 0.9611 | -0.4332 | FALSE |
| chr6 | 555 | 0.0617 | 1.0836 | 0.9311 | FALSE |
| chr7 | 454 | 0.0504 | 0.9663 | -0.3758 | FALSE |
| chr8 | 491 | 0.0546 | 1.0236 | 0.2625 | FALSE |
| chr9 | 415 | 0.0461 | 1.0666 | 0.7424 | FALSE |
| chr10 | 425 | 0.0472 | 1.0601 | 0.6699 | FALSE |
| chr11 | 451 | 0.0501 | 1.1986 | 2.2130 | FALSE |
| chr12 | 408 | 0.0453 | 0.9329 | -0.7476 | FALSE |
| chr13 | 287 | 0.0319 | 0.9077 | -1.0286 | FALSE |
| chr14 | 264 | 0.0293 | 0.9572 | -0.4768 | FALSE |
| chr15 | 231 | 0.0257 | 0.9691 | -0.3446 | FALSE |
| chr16 | 268 | 0.0298 | 1.0040 | 0.0444 | FALSE |
| chr17 | 217 | 0.0241 | 0.7757 | -2.4992 | FALSE |
| chr18 | 256 | 0.0284 | 0.9807 | -0.2146 | FALSE |
| chr19 | 202 | 0.0224 | 1.0004 | 0.0041 | FALSE |
| chr20 | 183 | 0.0203 | 1.0208 | 0.2318 | FALSE |
| chr21 | 136 | 0.0151 | 1.1801 | 2.0067 | FALSE |
| chr22 | 101 | 0.0112 | 0.9955 | -0.0500 | FALSE |
| Run2-B02 |  |  |  |  |  |
| **Chr** | **UA** | **%UA** | **Adjusted Relative Copy Number** | **Modified Z-score** | **Abnormal?** |
| chrMT | 14 | 0.0016 | 0.9989 | -0.0121 | FALSE |
| chrX | 261 | 0.0290 | 1.1639 | 1.8265 | FALSE |
| chrY | 19 | 0.0021 | 0.3111 | -7.6761 | TRUE |
| chr1 | 740 | 0.0822 | 0.9882 | -0.1311 | FALSE |
| chr2 | 750 | 0.0833 | 0.9717 | -0.3152 | FALSE |
| chr3 | 662 | 0.0736 | 1.0497 | 0.5533 | FALSE |
| chr4 | 594 | 0.0660 | 0.9775 | -0.2505 | FALSE |
| chr5 | 564 | 0.0627 | 1.0007 | 0.0077 | FALSE |
| chr6 | 520 | 0.0578 | 0.9989 | -0.0121 | FALSE |
| chr7 | 502 | 0.0558 | 1.0513 | 0.5713 | FALSE |
| chr8 | 485 | 0.0539 | 0.9948 | -0.0578 | FALSE |
| chr9 | 412 | 0.0458 | 1.0419 | 0.4670 | FALSE |
| chr10 | 453 | 0.0503 | 1.1118 | 1.2460 | FALSE |
| chr11 | 409 | 0.0454 | 1.0695 | 0.7747 | FALSE |
| chr12 | 431 | 0.0479 | 0.9697 | -0.3380 | FALSE |
| chr13 | 293 | 0.0326 | 0.9118 | -0.9830 | FALSE |
| chr14 | 298 | 0.0331 | 1.0631 | 0.7035 | FALSE |
| chr15 | 257 | 0.0286 | 1.0608 | 0.6779 | FALSE |
| chr16 | 227 | 0.0252 | 0.8367 | -1.8193 | FALSE |
| chr17 | 243 | 0.0270 | 0.8547 | -1.6190 | FALSE |
| chr18 | 255 | 0.0283 | 0.9612 | -0.4321 | FALSE |
| chr19 | 176 | 0.0196 | 0.8576 | -1.5867 | FALSE |
| chr20 | 200 | 0.0222 | 1.0977 | 1.0888 | FALSE |
| chr21 | 128 | 0.0142 | 1.0928 | 1.0344 | FALSE |
| chr22 | 107 | 0.0119 | 1.0377 | 0.4202 | FALSE |
| Run2-B03 |  |  |  |  |  |
| **Chr** | **UA** | **%UA** | **Adjusted Relative Copy Number** | **Modified Z-score** | **Abnormal?** |
| chrMT | 11 | 0.0012 | 0.7856 | -2.3895 | FALSE |
| chrX | 255 | 0.0283 | 1.1382 | 1.5397 | FALSE |
| chrY | 62 | 0.0069 | 1.0162 | 0.1805 | FALSE |
| chr1 | 756 | 0.0840 | 1.0105 | 0.1170 | FALSE |
| chr2 | 795 | 0.0883 | 1.0309 | 0.3446 | FALSE |
| chr3 | 599 | 0.0666 | 0.9506 | -0.5504 | FALSE |
| chr4 | 604 | 0.0671 | 0.9949 | -0.0572 | FALSE |
| chr5 | 527 | 0.0586 | 0.9359 | -0.7146 | FALSE |
| chr6 | 542 | 0.0602 | 1.0421 | 0.4692 | FALSE |
| chr7 | 534 | 0.0593 | 1.1193 | 1.3291 | FALSE |
| chr8 | 460 | 0.0511 | 0.9444 | -0.6198 | FALSE |
| chr9 | 392 | 0.0436 | 0.9922 | -0.0868 | FALSE |
| chr10 | 456 | 0.0507 | 1.1202 | 1.3391 | FALSE |
| chr11 | 415 | 0.0461 | 1.0862 | 0.9603 | FALSE |
| chr12 | 410 | 0.0456 | 0.9232 | -0.8553 | FALSE |
| chr13 | 324 | 0.0360 | 1.0091 | 0.1020 | FALSE |
| chr14 | 294 | 0.0327 | 1.0498 | 0.5549 | FALSE |
| chr15 | 224 | 0.0249 | 0.9254 | -0.8308 | FALSE |
| chr16 | 233 | 0.0259 | 0.8596 | -1.5644 | FALSE |
| chr17 | 227 | 0.0252 | 0.7991 | -2.2382 | FALSE |
| chr18 | 262 | 0.0291 | 0.9885 | -0.1283 | FALSE |
| chr19 | 164 | 0.0182 | 0.7998 | -2.2304 | FALSE |
| chr20 | 190 | 0.0211 | 1.0438 | 0.4875 | FALSE |
| chr21 | 162 | 0.0180 | 1.3843 | 4.2828 | TRUE |
| chr22 | 102 | 0.0113 | 0.9901 | -0.1103 | FALSE |
| Run2-B04 |  |  |  |  |  |
| **Chr** | **UA** | **%UA** | **Adjusted Relative Copy Number** | **Modified Z-score** | **Abnormal?** |
| chrMT | 10 | 0.0011 | 0.7525 | -2.7574 | FALSE |
| chrX | 532 | 0.0591 | 2.5022 | 16.7395 | TRUE |
| chrY | 12 | 0.0013 | 0.2073 | -8.8336 | TRUE |
| chr1 | 787 | 0.0874 | 1.1085 | 1.2090 | FALSE |
| chr2 | 797 | 0.0886 | 1.0891 | 0.9928 | FALSE |
| chr3 | 605 | 0.0672 | 1.0118 | 0.1311 | FALSE |
| chr4 | 560 | 0.0622 | 0.9720 | -0.3121 | FALSE |
| chr5 | 589 | 0.0654 | 1.1022 | 1.1391 | FALSE |
| chr6 | 512 | 0.0569 | 1.0374 | 0.4163 | FALSE |
| chr7 | 466 | 0.0518 | 1.0293 | 0.3262 | FALSE |
| chr8 | 464 | 0.0516 | 1.0038 | 0.0425 | FALSE |
| chr9 | 354 | 0.0393 | 0.9442 | -0.6217 | FALSE |
| chr10 | 448 | 0.0498 | 1.1597 | 1.7796 | FALSE |
| chr11 | 421 | 0.0468 | 1.1611 | 1.7955 | FALSE |
| chr12 | 393 | 0.0437 | 0.9326 | -0.7516 | FALSE |
| chr13 | 315 | 0.0350 | 1.0339 | 0.3775 | FALSE |
| chr14 | 245 | 0.0272 | 0.9219 | -0.8706 | FALSE |
| chr15 | 244 | 0.0271 | 1.0623 | 0.6940 | FALSE |
| chr16 | 246 | 0.0273 | 0.9564 | -0.4861 | FALSE |
| chr17 | 244 | 0.0271 | 0.9052 | -1.0566 | FALSE |
| chr18 | 242 | 0.0269 | 0.9621 | -0.4220 | FALSE |
| chr19 | 144 | 0.0160 | 0.7401 | -2.8965 | FALSE |
| chr20 | 177 | 0.0197 | 1.0246 | 0.2744 | FALSE |
| chr21 | 108 | 0.0120 | 0.9725 | -0.3062 | FALSE |
| chr22 | 85 | 0.0094 | 0.8695 | -1.4547 | FALSE |
| Run2-B05* | Normal male reference | |  |  |  |
| **Chr** | **UA** | **%UA** | **Adjusted Relative Copy Number** | **Modified Z-score** | **Abnormal?** |
| chrMT | 14 | 0.0016 | 1.0000 | NA | NA |
| chrX | 224 | 0.0249 | 1.0000 | NA | NA |
| chrY | 61 | 0.0068 | 1.0000 | NA | NA |
| chr1 | 748 | 0.0831 | 1.0000 | NA | NA |
| chr2 | 771 | 0.0857 | 1.0000 | NA | NA |
| chr3 | 630 | 0.0700 | 1.0000 | NA | NA |
| chr4 | 607 | 0.0674 | 1.0000 | NA | NA |
| chr5 | 563 | 0.0626 | 1.0000 | NA | NA |
| chr6 | 520 | 0.0578 | 1.0000 | NA | NA |
| chr7 | 477 | 0.0530 | 1.0000 | NA | NA |
| chr8 | 487 | 0.0541 | 1.0000 | NA | NA |
| chr9 | 395 | 0.0439 | 1.0000 | NA | NA |
| chr10 | 407 | 0.0452 | 1.0000 | NA | NA |
| chr11 | 382 | 0.0424 | 1.0000 | NA | NA |
| chr12 | 444 | 0.0493 | 1.0000 | NA | NA |
| chr13 | 321 | 0.0357 | 1.0000 | NA | NA |
| chr14 | 280 | 0.0311 | 1.0000 | NA | NA |
| chr15 | 242 | 0.0269 | 1.0000 | NA | NA |
| chr16 | 271 | 0.0301 | 1.0000 | NA | NA |
| chr17 | 284 | 0.0316 | 1.0000 | NA | NA |
| chr18 | 265 | 0.0294 | 1.0000 | NA | NA |
| chr19 | 205 | 0.0228 | 1.0000 | NA | NA |
| chr20 | 182 | 0.0202 | 1.0000 | NA | NA |
| chr21 | 117 | 0.0130 | 1.0000 | NA | NA |
| chr22 | 103 | 0.0114 | 1.0000 | NA | NA |
| Run2-B08 |  |  |  |  |  |
| **Chr** | **UA** | **%UA** | **Adjusted Relative Copy Number** | **Modified Z-score** | **Abnormal?** |
| chrMT | 12 | 0.0013 | 0.8578 | -1.3254 | FALSE |
| chrX | 250 | 0.0278 | 1.1170 | 1.5622 | FALSE |
| chrY | 62 | 0.0069 | 1.0172 | 0.4506 | FALSE |
| chr1 | 737 | 0.0819 | 0.9861 | 0.1038 | FALSE |
| chr2 | 796 | 0.0884 | 1.0332 | 0.6294 | FALSE |
| chr3 | 610 | 0.0678 | 0.9690 | -0.0863 | FALSE |
| chr4 | 580 | 0.0644 | 0.9563 | -0.2283 | FALSE |
| chr5 | 558 | 0.0620 | 0.9919 | 0.1687 | FALSE |
| chr6 | 507 | 0.0563 | 0.9758 | -0.0110 | FALSE |
| chr7 | 467 | 0.0519 | 0.9798 | 0.0340 | FALSE |
| chr8 | 521 | 0.0579 | 1.0707 | 1.0463 | FALSE |
| chr9 | 365 | 0.0406 | 0.9248 | -0.5792 | FALSE |
| chr10 | 457 | 0.0508 | 1.1237 | 1.6378 | FALSE |
| chr11 | 371 | 0.0412 | 0.9720 | -0.0534 | FALSE |
| chr12 | 402 | 0.0447 | 0.9061 | -0.7872 | FALSE |
| chr13 | 296 | 0.0329 | 0.9228 | -0.6008 | FALSE |
| chr14 | 253 | 0.0281 | 0.9043 | -0.8076 | FALSE |
| chr15 | 237 | 0.0263 | 0.9801 | 0.0374 | FALSE |
| chr16 | 305 | 0.0339 | 1.1264 | 1.6669 | FALSE |
| chr17 | 246 | 0.0273 | 0.8669 | -1.2244 | FALSE |
| chr18 | 394 | 0.0438 | 1.4880 | 5.6964 | TRUE |
| chr19 | 168 | 0.0187 | 0.8202 | -1.7450 | FALSE |
| chr20 | 188 | 0.0209 | 1.0338 | 0.6354 | FALSE |
| chr21 | 129 | 0.0143 | 1.1034 | 1.4116 | FALSE |
| chr22 | 89 | 0.0099 | 0.8648 | -1.2480 | FALSE |
| Run3-B01 |  |  |  |  |  |
| **Chr** | **UA** | **%UA** | **Adjusted Relative Copy Number** | **Modified Z-score** | **Abnormal?** |
| chrMT | 15 | 0.0017 | 1.0753 | 0.8386 | FALSE |
| chrX | 252 | 0.0280 | 1.1290 | 1.4377 | FALSE |
| chrY | 48 | 0.0053 | 0.7897 | -2.3434 | FALSE |
| chr1 | 771 | 0.0857 | 1.0344 | 0.3837 | FALSE |
| chr2 | 782 | 0.0869 | 1.0179 | 0.1994 | FALSE |
| chr3 | 626 | 0.0696 | 0.9972 | -0.0312 | FALSE |
| chr4 | 596 | 0.0662 | 0.9854 | -0.1629 | FALSE |
| chr5 | 530 | 0.0589 | 0.9447 | -0.6157 | FALSE |
| chr6 | 523 | 0.0581 | 1.0094 | 0.1043 | FALSE |
| chr7 | 504 | 0.0560 | 1.0604 | 0.6728 | FALSE |
| chr8 | 466 | 0.0518 | 0.9603 | -0.4424 | FALSE |
| chr9 | 386 | 0.0429 | 0.9807 | -0.2150 | FALSE |
| chr10 | 449 | 0.0499 | 1.1071 | 1.1938 | FALSE |
| chr11 | 442 | 0.0491 | 1.1612 | 1.7963 | FALSE |
| chr12 | 419 | 0.0466 | 0.9471 | -0.5899 | FALSE |
| chr13 | 303 | 0.0337 | 0.9473 | -0.5873 | FALSE |
| chr14 | 330 | 0.0367 | 1.1828 | 2.0368 | FALSE |
| chr15 | 250 | 0.0278 | 1.0367 | 0.4095 | FALSE |
| chr16 | 253 | 0.0281 | 0.9369 | -0.7030 | FALSE |
| chr17 | 228 | 0.0253 | 0.8057 | -2.1653 | FALSE |
| chr18 | 258 | 0.0287 | 0.9771 | -0.2556 | FALSE |
| chr19 | 160 | 0.0178 | 0.7833 | -2.4150 | FALSE |
| chr20 | 166 | 0.0184 | 0.9153 | -0.9433 | FALSE |
| chr21 | 136 | 0.0151 | 1.1665 | 1.8558 | FALSE |
| chr22 | 107 | 0.0119 | 1.0425 | 0.4741 | FALSE |
| Run3-B02 |  |  |  |  |  |
| **Chr** | **UA** | **%UA** | **Adjusted Relative Copy Number** | **Modified Z-score** | **Abnormal?** |
| chrMT | 12 | 0.0014 | 0.8795 | -1.3423 | FALSE |
| chrX | 237 | 0.0269 | 1.0857 | 0.9548 | FALSE |
| chrY | 55 | 0.0062 | 0.9252 | -0.8335 | FALSE |
| chr1 | 774 | 0.0878 | 1.0618 | 0.6887 | FALSE |
| chr2 | 799 | 0.0906 | 1.0634 | 0.7065 | FALSE |
| chr3 | 589 | 0.0668 | 0.9594 | -0.4529 | FALSE |
| chr4 | 602 | 0.0683 | 1.0177 | 0.1970 | FALSE |
| chr5 | 524 | 0.0594 | 0.9551 | -0.5009 | FALSE |
| chr6 | 517 | 0.0586 | 1.0202 | 0.2252 | FALSE |
| chr7 | 510 | 0.0578 | 1.0971 | 1.0823 | FALSE |
| chr8 | 480 | 0.0544 | 1.0114 | 0.1269 | FALSE |
| chr9 | 354 | 0.0401 | 0.9196 | -0.8956 | FALSE |
| chr10 | 407 | 0.0462 | 1.0261 | 0.2912 | FALSE |
| chr11 | 426 | 0.0483 | 1.1443 | 1.6082 | FALSE |
| chr12 | 389 | 0.0441 | 0.8990 | -1.1252 | FALSE |
| chr13 | 259 | 0.0294 | 0.8279 | -1.9173 | FALSE |
| chr14 | 264 | 0.0299 | 0.9675 | -0.3622 | FALSE |
| chr15 | 258 | 0.0293 | 1.0940 | 1.0472 | FALSE |
| chr16 | 270 | 0.0306 | 1.0223 | 0.2490 | FALSE |
| chr17 | 265 | 0.0301 | 0.9575 | -0.4738 | FALSE |
| chr18 | 243 | 0.0276 | 0.9409 | -0.6581 | FALSE |
| chr19 | 163 | 0.0185 | 0.8159 | -2.0514 | FALSE |
| chr20 | 196 | 0.0222 | 1.1051 | 1.1708 | FALSE |
| chr21 | 124 | 0.0141 | 1.0875 | 0.9753 | FALSE |
| chr22 | 101 | 0.0115 | 1.0062 | 0.0692 | FALSE |
| Run3-B03 |  |  |  |  |  |
| **Chr** | **UA** | **%UA** | **Adjusted Relative Copy Number** | **Modified Z-score** | **Abnormal?** |
| chrMT | 25 | 0.0028 | 1.8676 | 9.6674 | TRUE |
| chrX | 461 | 0.0512 | 2.1524 | 12.8410 | TRUE |
| chrY | 19 | 0.0021 | 0.3258 | -7.5132 | TRUE |
| chr1 | 713 | 0.0792 | 0.9969 | -0.0345 | FALSE |
| chr2 | 761 | 0.0846 | 1.0323 | 0.3596 | FALSE |
| chr3 | 638 | 0.0709 | 1.0591 | 0.6588 | FALSE |
| chr4 | 615 | 0.0683 | 1.0596 | 0.6644 | FALSE |
| chr5 | 542 | 0.0602 | 1.0068 | 0.0761 | FALSE |
| chr6 | 540 | 0.0600 | 1.0861 | 0.9590 | FALSE |
| chr7 | 501 | 0.0557 | 1.0985 | 1.0971 | FALSE |
| chr8 | 471 | 0.0523 | 1.0115 | 0.1279 | FALSE |
| chr9 | 376 | 0.0418 | 0.9955 | -0.0498 | FALSE |
| chr10 | 455 | 0.0506 | 1.1692 | 1.8852 | FALSE |
| chr11 | 415 | 0.0461 | 1.1362 | 1.5175 | FALSE |
| chr12 | 434 | 0.0482 | 1.0223 | 0.2483 | FALSE |
| chr13 | 298 | 0.0331 | 0.9709 | -0.3242 | FALSE |
| chr14 | 266 | 0.0296 | 0.9935 | -0.0719 | FALSE |
| chr15 | 226 | 0.0251 | 0.9767 | -0.2597 | FALSE |
| chr16 | 266 | 0.0296 | 1.0265 | 0.2958 | FALSE |
| chr17 | 224 | 0.0249 | 0.8249 | -1.9513 | FALSE |
| chr18 | 239 | 0.0266 | 0.9432 | -0.6326 | FALSE |
| chr19 | 150 | 0.0167 | 0.7652 | -2.6159 | FALSE |
| chr20 | 174 | 0.0193 | 0.9999 | -0.0015 | FALSE |
| chr21 | 94 | 0.0104 | 0.8402 | -1.7802 | FALSE |
| chr22 | 97 | 0.0108 | 0.9849 | -0.1681 | FALSE |
| Run3-B05 |  |  |  |  |  |
| **Chr** | **UA** | **%UA** | **Adjusted Relative Copy Number** | **Modified Z-score** | **Abnormal?** |
| chrMT | 17 | 0.0019 | 1.1972 | 2.4987 | FALSE |
| chrX | 253 | 0.0281 | 1.1136 | 1.5668 | FALSE |
| chrY | 43 | 0.0048 | 0.6950 | -3.0975 | FALSE |
| chr1 | 745 | 0.0828 | 0.9820 | 0.1004 | FALSE |
| chr2 | 758 | 0.0842 | 0.9693 | -0.0408 | FALSE |
| chr3 | 603 | 0.0670 | 0.9437 | -0.3264 | FALSE |
| chr4 | 620 | 0.0689 | 1.0071 | 0.3797 | FALSE |
| chr5 | 517 | 0.0574 | 0.9054 | -0.7532 | FALSE |
| chr6 | 517 | 0.0574 | 0.9803 | 0.0810 | FALSE |
| chr7 | 495 | 0.0550 | 1.0231 | 0.5590 | FALSE |
| chr8 | 498 | 0.0553 | 1.0082 | 0.3926 | FALSE |
| chr9 | 388 | 0.0431 | 0.9685 | -0.0503 | FALSE |
| chr10 | 470 | 0.0522 | 1.1386 | 1.8450 | FALSE |
| chr11 | 384 | 0.0427 | 0.9911 | 0.2019 | FALSE |
| chr12 | 421 | 0.0468 | 0.9349 | -0.4247 | FALSE |
| chr13 | 314 | 0.0349 | 0.9644 | -0.0952 | FALSE |
| chr14 | 275 | 0.0306 | 0.9683 | -0.0518 | FALSE |
| chr15 | 262 | 0.0291 | 1.0674 | 1.0524 | FALSE |
| chr16 | 270 | 0.0300 | 0.9823 | 0.1039 | FALSE |
| chr17 | 248 | 0.0276 | 0.8610 | -1.2482 | FALSE |
| chr18 | 263 | 0.0292 | 0.9785 | 0.0615 | FALSE |
| chr19 | 162 | 0.0180 | 0.7791 | -2.1601 | FALSE |
| chr20 | 194 | 0.0216 | 1.0509 | 0.8688 | FALSE |
| chr21 | 186 | 0.0207 | 1.5674 | 6.6236 | TRUE |
| chr22 | 97 | 0.0108 | 0.9285 | -0.4956 | FALSE |
| Run3-B06 |  |  |  |  |  |
| **Chr** | **UA** | **%UA** | **Adjusted Relative Copy Number** | **Modified Z-score** | **Abnormal?** |
| chrMT | 15 | 0.0017 | 1.0650 | 0.7242 | FALSE |
| chrX | 219 | 0.0243 | 0.9718 | -0.3142 | FALSE |
| chrY | 19 | 0.0021 | 0.3096 | -7.6932 | TRUE |
| chr1 | 756 | 0.0840 | 1.0046 | 0.0515 | FALSE |
| chr2 | 813 | 0.0903 | 1.0481 | 0.5364 | FALSE |
| chr3 | 667 | 0.0741 | 1.0524 | 0.5836 | FALSE |
| chr4 | 605 | 0.0672 | 0.9907 | -0.1034 | FALSE |
| chr5 | 538 | 0.0598 | 0.9499 | -0.5588 | FALSE |
| chr6 | 517 | 0.0574 | 0.9883 | -0.1308 | FALSE |
| chr7 | 510 | 0.0567 | 1.0628 | 0.6993 | FALSE |
| chr8 | 490 | 0.0544 | 1.0001 | 0.0013 | FALSE |
| chr9 | 400 | 0.0444 | 1.0066 | 0.0733 | FALSE |
| chr10 | 433 | 0.0481 | 1.0575 | 0.6406 | FALSE |
| chr11 | 390 | 0.0433 | 1.0148 | 0.1650 | FALSE |
| chr12 | 407 | 0.0452 | 0.9112 | -0.9899 | FALSE |
| chr13 | 320 | 0.0356 | 0.9909 | -0.1014 | FALSE |
| chr14 | 245 | 0.0272 | 0.8697 | -1.4515 | FALSE |
| chr15 | 255 | 0.0283 | 1.0474 | 0.5281 | FALSE |
| chr16 | 230 | 0.0256 | 0.8436 | -1.7427 | FALSE |
| chr17 | 275 | 0.0306 | 0.9625 | -0.4179 | FALSE |
| chr18 | 265 | 0.0294 | 0.9940 | -0.0669 | FALSE |
| chr19 | 189 | 0.0210 | 0.9164 | -0.9314 | FALSE |
| chr20 | 196 | 0.0218 | 1.0705 | 0.7851 | FALSE |
| chr21 | 135 | 0.0150 | 1.1469 | 1.6371 | FALSE |
| chr22 | 111 | 0.0123 | 1.0712 | 0.7934 | FALSE |
